# Exposure to Crimean-Congo Hemorrhagic Fever Virus in Wild Ungulates in the Basque Country, Northern Iberian Peninsula

**DOI:** 10.1155/tbed/8553577

**Published:** 2024-12-10

**Authors:** Aitor Cevidanes, Jesús F. Barandika, Gorka Aduriz, Ana Hurtado, Ana L. García-Pérez, Marta Barral

**Affiliations:** Department of Animal Health, NEIKER-Basque Institute for Agricultural Research and Development, Basque Research and Technology Alliance (BRTA), Derio, Bizkaia, Spain

**Keywords:** arbovirus, Crimean-Congo hemorrhagic fever, tick, ungulates, zoonosis

## Abstract

Crimean-Congo hemorrhagic fever virus (CCHFV) causes a serious human tick-borne disease. In animals, CCHFV infections are mainly subclinical. The circulation of the virus has received little attention in areas where the main vector (*Hyalomma* spp.) is not considered to be present or established (e.g., the Northern Iberian Peninsula). The presence of antibodies against CCHFV was evaluated in sera collected from 1190 wild boars, 36 red deer, and 36 roe deer in the Basque Country (Northern Iberian Peninsula) in 2014–2019. Antibodies were found in the three wild ungulate species with an overall prevalence of 2.5%. The highest seroprevalence was found in red deer (22.2%) and in the southwest province: Araba (8.6%). The presence of antibodies against CCHFV in wild ungulates reported in this study could be due to an underestimated presence of *Hyalomma* ticks, the sporadic exposure to infected *Hyalomma* ticks transported by animals (e.g., migratory birds), or the role of other tick species in the virus's circulation. The detection of exposed animals since 2014 suggests that the circulation of the virus beyond the southwestern regions of the Iberian Peninsula could have been more widespread than previously thought.

## 1. Introduction

Crimean-Congo hemorrhagic fever (CCHF) is a viral tick-borne disease with major human health implications. Its etiological agent, the Crimean-Congo hemorrhagic fever virus (CCHFV), is considered the most relevant tick-borne virus in the world, due to its distribution, severity, and mortality [1]. CCHFV is an *Orthonairovirus* (family Nairoviridae) mainly transmitted by ticks of the genus *Hyalomma* and is currently distributed in Africa, Asia, and Europe [2, 3]. In Europe, its presence is restricted mainly to Southeastern Europe and the Mediterranean Basin [4]. The time since CCHFV has been circulating in southwestern Europe is unknown. No previous evidence of the presence of the virus in the Iberian Peninsula existed until the 1980s, when antibodies against the virus were detected in two people in Portugal [5]. In Spain, the first detection was made in *Hyalomma lusitanicum* ticks retrieved from red deer (*Cervus elaphus*) in 2010 [6]. Since then, different studies have shown the presence of the virus in ticks removed from ungulates [7, 8]. The first human case reported in Spain was in 2016 [9]; however, a recent retrospective study traced the first human case back to 2013 [10]. Since 2013, 17 cases have been reported in Spain [11, 12]. The bite of an infected tick is the most common cause of human CCHF cases [2]. However, exposure to the blood and tissues of infected animals can also cause CCHF in humans [2].

In nature, CCHFV is maintained by enzootic cycles through vertical and horizontal transmission involving vertebrates and ticks [1]. In ticks, the transmission and maintenance of CCHFV occur via transovarial transmission and transstadial survival of the virus. This is why ticks are considered both vectors and reservoirs of the virus [13]. The distribution of the virus is closely associated with the presence of the main vectors, ticks of the genus *Hyalomma* [13]. Thus, changes in the spatial distribution of the vectors could drive CCHFV circulation changes.

Vertebrates play an essential role in CCHF epidemiology because, in addition to their capacity to transmit CCHFV to ticks, they maintain the tick populations by blood feeding and can carry ticks over large distances [14]. The role that migratory birds play in the dispersal of *Hyalomma* and CCHFV-infected ticks is well-known [15–17]. The Northern Atlantic regions of the Iberian Peninsula, in general, are considered a *Hyalomma*-free zone, but in the southern part of the Basque Country, where this study has been conducted, a few specimens of *Hyalomma marginatum* have been found in domestic ungulates (unpublished data). It should be noted that, in the regions bordering with the Basque Country on the south, specimens of *H. marginatum* have been found in wild birds, vegetation, and cattle [18, 19]. The Basque Country receives large numbers of passing migratory wild birds from Africa and includes hotspots of great relevance in the migratory routes [20]. The finding of wildlife exposed to the virus in areas without a clear presence of *Hyalomma* ticks [21–23] gave more weight to the hypotheses of sporadic introductions by migratory birds as well as the possibility that *Hyalomma* presence is underestimated in some places. Additionally, other species of ticks may also act as potential vectors, but only ticks of the genus *Hyalomma* are able to maintain an endemic focus of the virus after its introduction [24]. Because CCHFV-infected animals do not show symptoms and viremias are transient, serological studies are an important source of information to monitor areas with virus transmission and identify exposed species [1, 14].

Nonetheless, the circulation of CCHFV has received little attention in areas where *Hyalomma* is not considered to be present or established. For this reason, this study aims to evaluate the presence of antibodies against CCHFV in wild ungulates of the Basque Country, a region in the northern part of the Iberian Peninsula without previous reports of CCHFV exposure and where *Hyalomma* ticks are not considered to have been fully established.

## 2. Material and Methods

### 2.1. Study Area and Sampling

**Figure 1:**
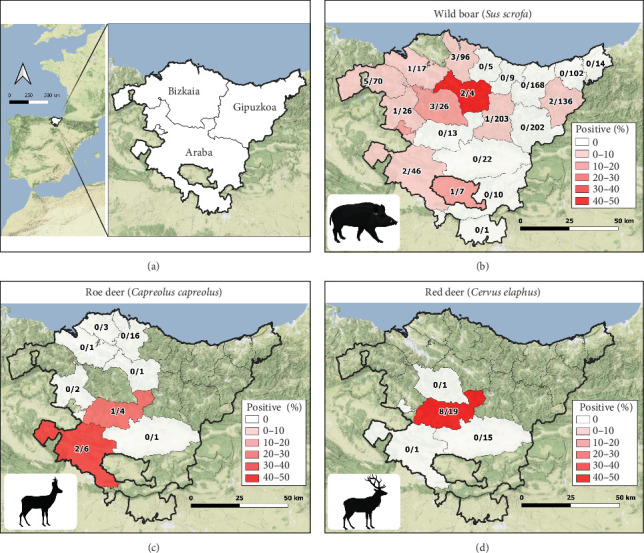
Localization of the Basque Country and the three provinces (A). The number of seropositive wild boar (B), roe deer (C), and red deer (D) specimens per total analyzed per county. Increasing intensity of the gradation of the red color in the counties indicates a higher percentage of seropositive animals, according to the legend. Non-colored counties correspond to non-sampled counties. Three and ten wild boars from Gipuzkoa and Bizkaia, respectively; and two roe deer from Bizkaia lacking information regarding county of origin information resulted seronegative.

The survey was carried out in the Basque Country, in the Northern Iberian Peninsula (Figure 1A), a 7234 km^2^ region that includes three provinces (Araba, Bizkaia, and Gipuzkoa) and 21 counties and that is divided into two simplified bioclimatic regions [25]: the Atlantic climate in the northern areas and the Continental Mediterranean climate in the south. The Atlantic climate is characterized by abundant precipitation and mild summers and winters, while the Continental Mediterranean climate is characterized by hot summers and cold winters. European wild boar (*Sus scrofa*), roe deer (*Capreolus capreolus*), and red deer (*C. elaphus*) are the most common wild ungulate species in the Basque Country. Wild boar and roe deer are extensively distributed throughout the territory. However, red deer are limited to relatively small geographical areas, mainly to the area of Mount Gorbea (Araba) and Karranza (Bizkaia) [26]. There is almost no fencing of wildlife habitats, and artificial management of wildlife is scarce, allowing them to come into contact with domestic ruminants not only when grazing on communal pastures but also when grazing on pastures surrounding farms [27].

Serum samples from 1190 wild boars (*S. scrofa*), 36 red deer (*C. elaphus*), and 36 roe deer (*C. capreolus*) collected in the period 2014–2019 within the context of a wildlife health surveillance program in the Basque Country were included in the study. Most samples (97%) were collected from hunted animals; in all cases, hunting had the authorization of local authorities and was carried out in agreement with Spanish and European regulations. Sera were maintained at −20°C until processing.

### 2.2. Serological Analyses

The presence of antibodies against CCHFV in ungulate serum samples was tested using ID Screen CCHF Double Antigen Multispecies enzyme-linked immunosorbent assay (ELISA) kit (Innovative Diagnostics [ID]-Vet, https://www.id-vet.com) following the manufacturer's instructions. A serum sample was considered positive when the S/P (%) cutoff value was above 30. This test has a sensitivity of 98.9% and a specificity of 100% [28]. This ELISA test has been previously used in different domestic and wild animal species in Europe [23, 29–33].

### 2.3. Statistical Analysis

Statistical analyses were performed using the R statistical software version 3.0.1 [34]. Observed prevalence and 95% confidence interval (CI) were estimated. Differences in the prevalence (represented as occurrence for statistical analysis) according to wild ungulate species (wild boar, red deer, and roe deer), province (Araba, Bizkaia, and Gipuzkoa), season (spring, summer, autumn, and winter), and year (2014–2019) were evaluated first by bivariate nonparametric tests (chi-square test or Fisher's exact test) and those variables with *p*-values < 0.20 were included in the multivariate binomial logistic regression models. Seven wild boar samples from Treviño County (belonging in the province of Burgos but geographically located within the province of Araba) were included in the Araba province for analyses purposes. Due to the lack of data for some samples, the overall associations between seropositivity and sex (male/female) and age (adult/juvenile) were analyzed for 1113 wild ungulates. The best model was selected based on Akaike Information Criterion corrected (AICc) for sample size and odds ratios were calculated based on the generalized linear models (GLMs).

The comparison between S/P (%) values of the seropositive animals (above the cutoff value of 30) and ungulate species was evaluated using Kruskal–Wallis nonparametric rank test.

## 3. Results

An overall seroprevalence of 2.5% (32/1262; 95% CI, 1.6–3.4) was found. Antibodies against CCHFV were found in the three wild ungulate species and in the three provinces. Seropositive animals were found in all the years except in 2018. Summer was the only season without any seropositive animals. Seroprevalence by wild ungulate species, province, year, and season are shown in Table 1. Significantly higher seroprevalences were found in Araba (8.6%) and Bizkaia (5.6%) when compared with Gipuzkoa (0.3%). The highest seroprevalence was found in red deer (22.2%), while 8.3% of the roe deer and 1.7% of the wild boars were found to be seropositive.

The bivariate analysis found a significant association for species, province, year, and season (Table 2), and these variables were included in the multivariate analysis. No significant association was found between seropositivity and sex or age. The best multivariate logistic regression model (that retained species, province, and year variables) shows that there are 17 and 19 times more chances of finding a seropositive wild ungulate in Araba and Bizkaia, respectively, than in Gipuzkoa (Table 2). Moreover, red deer are five times more likely to be seropositive to CCHFV when compared to wild boar (Table 2). In 2016, the probability to find a seropositive wild ungulate was six times higher when compared with 2014.

**Figure 2:**
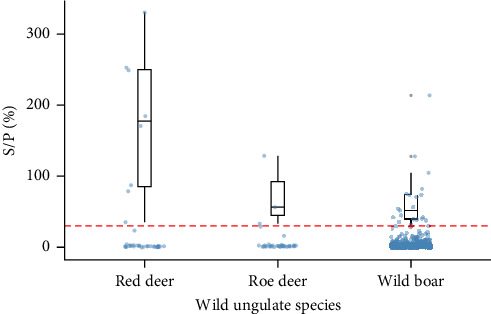
S/P (%) values of the ELISA by ungulate species. Boxplot corresponds to values above the cutoff value of 30 (red dashed line).

The 21 seropositive wild boars were found in 10 different counties, mainly in the Western Basque Country (in Araba and Bizkaia provinces) in all the years except 2018 (Figure 1B). All seropositive red deer were spatially aggregated but not temporally. Specifically, the eight seropositive deer were from different sites and years of Gorbeialdea County (Figure 1D). Three roe deer were found to be seropositive, and all three were found in the northwestern part of Araba province (two in 2015 and one in 2016) (Figure 1C). Red deer showed a higher S/P (%) value among the positive animals compared with roe deer and wild boars (*p*-value = 0.02) (Figure 2).

## 4. Discussion

Wild animals exposed to CCHFV were found in the Northern Iberian Peninsula. In general terms, a low exposure for CCHFV was found in wild ungulates in the Basque Country. The overall prevalence found in this study (2.5%) is similar to the 2% seroprevalence reported in wild animals in other areas of the Iberian Peninsula with scarce presence of *Hyalomma* [21]. In the Eastern Iberian Peninsula, recent studies found that seropositive wild ungulates were clustered near wetlands of great importance for bird migration [23, 35]. In our study, spatial aggregation of CCHFV exposure was also found for red deer (all the exposed animals in one county) and roe deer (all the exposed animals in two adjacent counties), while exposed wild boars were found in different counties (mainly in the western counties). It should be taken into account that the sample size of red deer and roe deer was small and that the distribution of red deer in the Basque Country is limited to relatively small geographical areas [26]. For this reason, in this case, spatial aggregation could reflect favorable conditions for the presence of the vector or a host-specific association. Regarding the different situations observed between years, the low number of seropositive animals detected in the last 3 years of the study might be due to the lower number of animals analyzed and not necessarily reflect a real decrease in prevalence.

Our study showed a clearly higher probability of exposure of red deer to CCHFV compared to roe deer and wild boar. The reasons for the observed differences between species are unknown, but in Southwestern Spain red deer was the most common host of CCHFV-infected ticks [36]. In addition, in our study, seropositive red deer had higher S/P (%) values than the other species. Nevertheless, since the test used is not designed for antibody quantification, care should be taken not to over-interpret these results. Red deer has been considered a good indicator of CCHFV in the Iberian Peninsula due to its abundance and distribution, gregarious behavior, and its importance as host of *Hyalomma* and other ticks [22]. However, this species would not be a good indicator for the spatial distribution of the CCHFV in the Basque territory due to its limited distribution. In Spain, 61% of the surveyed populations of red deer had at least one seropositive animal, but prevalence was higher (up to 88%) in the southwestern part of the Iberian Peninsula, whereas the northern part of the Iberian Peninsula was included in the cluster of low-risk areas [22].

Although *H. marginatum* is considered the most relevant species for CCHFV transmission in Europe [13], *H. lusitanicum* also plays a predominant role in the circulation of CCHFV in Spain [7, 36]. However, in the northern areas of the Iberian Peninsula, *Hyalomma* ticks infesting wild ungulates have never been reported [37–39]. Due to the increasing population densities and dispersion of wild boar [26], one of the natural hosts of *H. lusitanicum* [39, 40], it is believed that this tick species may be expanding its distribution range [36], thus changing the circulation scenario of CCHFV. The CCHFV seropositivity in wild ungulates revealed in this study could either reflect that the presence of *Hyalomma* ticks in the region has been underestimated or be the consequence of the sporadic exposure to unestablished *Hyalomma* infected ticks transported by animals [16]. Livestock transportation from endemic areas has also been pointed out as the source of CCHFV-infected *Hyalomma* spp. ticks [41]. Wild birds migrating from Africa is one of the most accepted hypotheses among the scientific community for the transportation of *Hyalomma* spp. immature stages and CCHFV-infected ticks [15–17, 42–44].

On the other hand, although these sporadic introductions of ticks are taking place, the immature stages need favorable environmental conditions at the destination to complete their life cycle and lead to established populations. For this reason, suitable environmental conditions are essential for the expansion and establishment of the CCHFV and its vectors. It has been predicted that climate change could displace favorable habitats for *Hyalomma* toward the northern regions of the Iberian Peninsula and Europe [45, 46]. Indeed, in the last years, permanent populations are found in southern continental France [43], with CCHFV detected in resident *H. marginatum* populations retrieved from horses and cattle in 2022 and 2023, confirming its circulation [47]. Therefore, climate change could be a driver for the expansion and the establishment of *Hyalomma* in the Basque Country.

Some studies reported the molecular detection of CCHFV also in tick species other than *Hyalomma* spp. from wildlife in the Iberian Peninsula [7, 36]. However, the detection of a pathogen in arthropods feeding on hosts provides no evidence of vector competence, which needs to be demonstrated by experimental studies [44]. In addition to ticks of the genus *Hyalomma*, *Rhipicephalus bursa* is also considered relevant in the transmission of CCHFV [13]. Furthermore, transovarial transmission has also been confirmed for *Dermacentor marginatus* [13]. Recently, the virus was detected in questing non-*Hyalomma* spp. ticks, primarily in *D. marginatus*, in Northwest Spain [32]. This area was visited by a CCHF patient for a walk before the onset of symptoms, adding evidence of the possible role of non-*Hyalomma* spp. ticks in the eco-epidemiological dynamics of CCHFV. In fact, *R. bursa* and *D. marginatus* have been found in wild ungulates and vegetation in the Basque Country [37, 38, 48]. Although their presence is not enough to confirm the role of those tick species in maintaining the virus, the importance that these species of ticks could have in this region cannot be completely ruled out. The role of wild birds in CCHF dynamics as tick hosts and carriers of infected ticks to new areas is recognized, but their importance as a reservoir and amplifier of CCHFV is considered low since experimental studies failed to produce viremia in avian species [14]. However, for some tick-borne viruses, transmission between cofeeding ticks can take place in the absence of detectable viremia, also known as “non-viremic transmission” [49, 50]. Thus, for viruses with “cofeeding” transmission mechanisms, host species without apparent viremia should not be completely ruled out in their role of virus maintenance and transmission.

A recent study found high CCHFV exposure in wild and domestic suid populations in endemic areas of Southwestern Spain [51], where the highest prevalence of CCHFV exposure in red deer also occurred [22]. Wild ungulates have been used as predictors of exposure risk. Recent studies have highlighted various high-risk foci for CCHFV exposure across Spain based on wild ungulate seroprevalence. The spatial projection of wild boar-based models identified high-risk areas in most of Western and Southwestern Iberia, as well as recently confirmed risk foci in Eastern Spain [31]. The highest predicted exposure risk in the southwest of the Iberian Peninsula was similar for wild boar and red deer, with slight differences probably due to the different spatial distribution patterns of both ungulate species [22, 31]. The wide circulation of CCHFV in wildlife and ticks does not seem to reflect the low number of human cases in Spain. It has been demonstrated that exposure risk patterns based on predictive wild animal–tick–virus models do not always necessarily agree with patterns of human CCHF cases [52]. Therefore, the factors that affect the encounters between *Hyalomma* ticks and humans need to be deeply studied. Although transmission to humans occurs mainly through tick bites, direct contact with fluids or tissues of viremic animals is also a route of transmission. The duration of the viremia in wild ungulates is unknown and human exposure to the virus, in general, can be considered low in Basque Country. However, the importance of biosecurity measures in the management of wild ungulates found dead or hunted, especially deer, should be highlighted.

In conclusion, this study extends the exposure map to CCHFV of wild animals in the Iberian Peninsula. The detection of exposed wild boars and red deer since 2014 suggests that the circulation of CCHFV in regions of the Iberian Peninsula other than the southwestern area could have been more widespread than previously thought. Molecular detection of the virus in this region coupled with the corresponding phylogenetic analysis and the study of domestic ungulates would help to understand the origin and dynamics of CCHFV in the northern part of the Iberian Peninsula. On the other hand, the distribution ranges of ticks of the genus *Hyalomma* are expected to expand northward due to climate change. Thus, continuous surveillance of both domestic and wild animals in relation to CCHFV as well as monitoring and in-depth evaluation of the presence of *Hyalomma* ticks throughout the territory of the Basque Country would help to better understand the potential spatial risk of CCHF in this region. For all these reasons, wildlife health and entomological surveillance are key for understanding and predicting the risks associated with emerging pathogens from a One Health approach.

## Figures and Tables

**Table 1:** Observed seroprevalences in wild ungulates by species, province, year, and season.

Variables	Wild boar (*Sus scrofa*)		Red deer (*Cervus elaphus*)		Roe deer (*Capreolus capreolus)*		Overall	
	*N*	% (CI)	*N*	% (CI)	*N*	% (CI)	*N*	% (CI)
Overall	1190	1.76 (1.02–2.51)	36	22.22 (8.64–35.80)	36	8.33 (0.00–17.36)	1262	2.54 (1.67–3.40)
Province								
Gipuzkoa	841	0.36 (0.00–0.76)	0	—	0	—	841	0.36 (0.00–0.76)
Bizkaia	223	6.28 (3.09–9.46)	1	0	23	0	247	5.67 (2.78–8.55)
Araba	126	3.17 (0.11–6.24)	35	22.86 (8.95–36.77)	13	23.08 (0.17–45.98)	174	8.62 (4.45–12.79)
Year								
2014	539	0.37 (0.00–0.88)	7	42.86 (6.20–79.52)	13	0	559	0.89 (0.11–1.67)
2015	328	1.83 (0.38–3.28)	10	20.00 (0.00–44.79)	16	12.50 (0.00–28.70)	354	2.82 (1.10–4.55)
2016	222	4.50 (1.78–7.23)	5	40.00 (0.00–82.94)	7	14.29 (0.00–40.21)	234	5.56 (2.62–8.49)
2017	27	7.41 (0.00–17.29)	8	12.50 (0.00–35.42)	0	—	35	8.57 (0.00–17.85)
2018	45	0	1	0	0	—	46	0
2019	29	3.45 (0.00–10.09)	5	0	0	—	34	2.94 (−2.74 to 8.62)
Season								
Spring	64	4.69 (0.00–9.87)	1	100	16	6.25 (0.00–18.11)	81	7.41 (1.70–13.11)
Summer	76	0	3	0	0	—	79	0
Autumn	620	0.81 (0.10–1.51)	14	35.71 (10.61–60.81)	4	0	638	1.57 (0.60–2.53)
Winter	430	3.02 (1.40–4.64)	18	11.11 (0.00–25.63)	16	6.25 (0.00–18.11)	464	3.45 (1.79–5.11)

*Note:* %, prevalence; CI, 95% confidence interval; *N*, total number of analyzed animals.

**Table 2:** *p*-Values of bivariate analysis (Fisher's exact test) and the summary of the best multivariate logistic regression model.

Variables	Bivariate analysis	Multivariate analysis			
	*p*-Value	Estimate ± SE	*Z*-value	*p*-Value	OR (95% CI)
Intercept	—	−6.45 ± 0.70	−9.18	<0.001*⁣*^*∗*^	—
Province	*p* < 0.001	—	—	—	—
Gipuzkoa	—	Reference	—	—	—
Bizkaia	—	2.96 ± 0.65	4.51	<0.001*⁣*^*∗*^	19.34 (6.02–86.20)
Araba	—	2.84 ± 0.71	3.99	<0.001*⁣*^*∗*^	17.20 (4.58–82.85)
Species	*p* < 0.001	—	—	—	—
Wild boar	—	Reference	—	—	—
Red deer	—	1.75 ± 0.60	2.91	0.003*⁣*^*∗*^	5.77 (1.77–19.21)
Roe deer	—	0.31 ± 0.67	0.47	0.63	1.37 (0.30–4.53)
Year	*p* < 0.001	—	—	—	—
2014	—	Reference	—	—	—
2015	—	0.68 ± 0.56	1.19	0.23	1.97 (0.67–6.57)
2016	—	1.79 ± 0.55	3.23	0.001*⁣*^*∗*^	6.01 (2.13–19.61)
2017	—	0.58 ± 0.80	0.73	0.46	1.80 (0.32–8.40)
2018	—	−14.98 ± 948.93	−0.01	0.98	0.00
2019	—	−0.43 ± 1.13	−0.38	0.70	0.64 (0.03–4.43)
Season	*p* < 0.01	Included in the multivariate analysis but not selected in the best model			

*Note:* The asterisk indicates a significant *p*-value of the multivariate analysis (below 0.05).

Abbreviation: SE, standard error.

## Data Availability

The data that support the findings of this study are available from the corresponding author upon request.
